# Traumatic Synovitis of the Knee

**Published:** 1907-06-01

**Authors:** 


### Points in Surgery.

### TRAUMATIC SYNOVITIS OF THE KNEE.

### I. DIAGNOSIS.

In this article it is proposed to deal with the ordinary type of case in which a pure traumatic synovitis follows an injury to the knee-joint. For clearness and brevity, the very severe injuries associated with fracture of bone or extensive laceration of ligaments will be excluded, and the various forms of infective arthritis, in the production of which trauma sometimes plays a part, will be mentioned no more than incidentally.

At the first thought there may appear to be but little scope for discussion with regard to the diagnosis of traumatic synovitis. The subject appears so simple. Yet this apparent simplicity is continually producing a rich crop of errors. As a matter of fact, much care and deliberation are required, for there are numerous pitfalls for the rash and unwary diagnostician. Thus, to take an imaginary case, a patient seeks medical advice because he has a swollen and painful knee, and he attributes the condition to an injury received a week previously in getting out of a dogcart. He has obvious synovitis; he has had an injury; therefore, argues the incautious one, he has traumatic synovitis. So the patient is advised and treated accordingly. A week later he complains of pain and swelling in one of the testicles, and it becomes clear that he has gonorrhœal epididymitis, and this is followed by the revelation that trauma, if it played any part at all in the inflammation of his knee, was quite subsidiary to gonorrhœal infection. Examination of the urine in such a case would almost certainly reveal the white shreds of gonorrhœa, and so lead to a correct interpretation of the joint affection. The writer has himself seen osteo-arthritis, tuberculosis, syphilis, gonorrhœa, and rheumatism of the knee, each buried away—for a time—beneath a diagnosis of traumatic synovitis.

As to the lesion which is present in any particular case of traumatic arthritis, it is not possible to do more than make a good guess. There may be laceration of a semilunar cartilage, especially the inner one, or a synovial fringe may have been pinched between the femur and tibia, or some other damage may have been done. However, apart from this doubt about the actual lesion, the cases form a group sufficiently homogeneous for collective consideration.

#### Examination of the Patient.

The clinical investigation must be systematic and thorough. Mere examination of the injured joint is not sufficient; a complete survey of the case is essential. As to the history, there ought to be a perfectly clear and definite account of a sudden painful injury to a knee in which nothing abnormal had been felt up to the moment of the accident, the injury being followed within a few hours by swelling of the joint. If the history of trauma is indistinct, if the patient thinks he hurt his knee, but is not quite certain about it, or if the swelling of the joint did not appear until a week or so after the supposed cause, suspicion should arise that the case may be one of infective arthritis, in the causation of which trauma may or may not have played a part. Locking of the knee—that is to say, inability to extend the leg immediately after the accident—is good evidence that a semilunar cartilage has been damaged. The patient's account of the local affection having been elicited, he should be asked if he has had previous trouble with his knee, if other joints are or have been affected, and also if he is in good health and free from any constitutional trouble. If his replies are satisfactory and confirmed by the usual general examination, attention may be directed to the injured part. For this purpose both lower limbs should be made bare, so that fair comparisons can be drawn. On looking at the affected joint one expects to find it in a position of slight flexion and distended with fluid. Palpation reveals a moderate increase of surface temperature, as compared with the sound side, and there is some tenderness; but there should be little if any wasting of the muscles of the injured side. In the absence of an obvious hæmatoma, pitting on pressure is suggestive of either fracture or infection; and if the skin be dusky or reddened one may suspect that something more than simple traumatic synovitis is present.

On handling the joint in such a manner as to obtain fluctuation, the capsule of the joint is felt to be normally thin and flexible, so that the sensation of fluctuation is easily obtained and the fluid seems close beneath the fingers. But in some infective conditions, especially in gonorrhœal and pneumococcal arthritis, there is sometimes much periarticular effusion, which renders the capsule hard and stiff, and so obscures the fluctuation of fluid within. And in syphilitic and tuberculous synovitis the synovial membrane may be swollen and "pulpy," and this, too, obscures fluctuation.

When the knee-joint is distended with fluid the patella is pushed away from the femur, with which normally it is in contact. If the effusion be of the kind which is poured out in response to trauma, the patella ought to be freely movable from side to side, and, when pressed backwards, the contact between the patella and femur should be felt as a clear, unmuffled tap. In the presence of periarticular effusion the patella cannot be so freely moved from side to side, and if there be pulpy swelling of the synovial membrane, the tap of the patella against the femur will be muffled. In advanced cases of infection the patella may be fixed to the femur by organising granulation tissue, but such advanced instances of infective arthritis are hardly liable to be confused with traumatic cases.

Though tedious and difficult to describe, these manipulative examinations of the joint are quickly and easily performed, and are capable of conveying important information.

There is one more point. Pressure over the internal semilunar cartilage is usually painful, and this has been supposed to indicate injury to the cartilage. It has no such meaning, for tenderness at this point is present in most cases of arthritis.

Only to the dull or inexperienced will all the above suspicions and precautions appear excessive. A few days ago the writer saw a case which had at first been diagnosed as traumatic synovitis of the knee turn out to be an acute suppurative arthritis; and in another recent case a knee-joint was operated on for the removal of a damaged semilunar cartilage, whereas tuberculous arthritis was discovered. In the latter case there was no doubt whatever about the history of sudden severe trauma; and in the former also, though the history was not so definite, the trouble was believed to have originated in a sprain. These and numerous other cases demonstrate that without the utmost care and persevering caution mistakes in diagnosis can hardly be avoided.

